# Perceived motivators, knowledge, attitude, self-reported and intentional practice of female condom use among female students in higher training institutions in Dodoma, Tanzania

**DOI:** 10.1186/s40834-022-00208-6

**Published:** 2023-02-08

**Authors:** Getrude W. Shitindi, Walter C. Millanzi, Patricia Z. Herman

**Affiliations:** Department of Nursing Management and Education, The University of Dodoma, Dodoma, Tanzania

**Keywords:** Condom use, Female condom, Female student, University of Dodoma, Tanzania

## Abstract

**Background:**

Unplanned pregnancies and sexually transmitted infections (STIs) Human Immunodeficiency Virus (HIV) inclusive, have remained to be a significant public health challenges among young people, especially across middle and low-income countries. Self-efficacy on the right ways of using condoms appears to be effective against the aforementioned health-related problems. However, most women of reproductive age tend not to use them when they are using highly effective contraceptives such as implants, and/or injectable hormones. It is unknown whether the uptake of female condoms among young girls is significantly high or not. Examining female students’ perceived motivators, knowledge, attitudes, and self-reported and intentional female condom practice in training institutions seems to be a key precursor in addressing the challenge.

**Methods:**

The institutional-based analytical cross-sectional survey in a quantitative research approach was adopted to study 384 randomly selected female students from higher training institutions, in Tanzania. Interviewer-administered structured questionnaires from previous studies were the main data collection tools. Data analysis was done using a statistical package for social science (SPSS) with the strength of statistical limits set at a 95% confidence interval and a 5% significance level.

**Results:**

The response rate of the study was 100% with 24 ± 2.122 years respondents’ mean age while and 32.7% of them were in the third year of their studies. Only 6.2% of the respondents reported having used female condoms while 26.3% of them have not ever seen packages of female condoms. The minority of the respondents (21.7%, 23.3%, and 9.4%) demonstrated good knowledge, positive attitude, and intention to use female condoms respectively. Their age, accommodation, marital status, peer groups, previous training, knowledge, and attitude related significantly to their intentional practices of female condom use (*p* < 0.05).

**Conclusion:**

Given the existing governmental and non-governmental efforts that advocate awareness creation and the uptake of various contraceptives, this study has found that knowledge, attitude, self-reported and intentional practices of female students admitted in higher training institutions within Dodoma region, Tanzania is low. Institutionalized educational programs on sexual and reproductive health matters may need to be prioritized to increase the availability, accessibility, and uptake of female condoms among female students in higher training institutions.

## Background

A call on countries by the sustainable development goal 3 (SDG3) target 3.7 emphasizes that the target of universal access to sexual and reproductive healthcare services including family planning, information and education, and the integration of reproductive health into national policies and programs must be achieved by 2030 [1]. National health strategic plans across nations show clear commitments to ensure that services are available and accessed easily by both males and females around the globe [2]. Availability and accessibility to the widest possible range of safe family planning services are believed to promote free and informed choices for appropriate and preferred contraceptive methods [3]. However, unplanned pregnancies and sexually transmitted infections HIV inclusive have remained to be a significant public health challenge from the early onset of unsafe sexual behaviors, particularly in young people especially across middle and low-income countries [4, 5].

Reports uncover that 57 and 48% of young girls and boys have claimed to have had unsafe sexual intercourse by the age of 18 years. Unsafe sexual behaviours have been linked with the incidences of STIs/HIV and unplanned pregnancies [6, 7]. Evidence shows that approximately 250,000 young people of school age are newly infected with HIV out of which an estimated 182, 599 (73%) are from Sub-Saharan African regions [8]. Nevertheless, 104, 400 out of 1,800,000 young people living with HIV in the globe are living in Tanzania whereas 57, 000 are young girls [9]. On the other hand, an estimated 21 million girls become pregnant every year and 2.5 million of them become mothers by the age of 16 years with a prevalence of 21.5% in East Africa Tanzania inclusive and 9.2 in Northern Africa. The prevalence of unplanned pregnancies among young girls in Tanzania is approximately at the second position (22.8%) with 27.0% being underage pregnancies to 23.8% in Uganda, 7.3% in Rwanda, and 12.4% in Ethiopia [10].

The global trend of STIs/HIV and unplanned pregnancies may imply that out of 1.9 billion women of reproductive age (15-49 years), 1.1 billion have a need and right for family planning services. Available statistical information indicates that an estimated 922 million women living in the globe by 2019 use contraceptives [11]. However, 790 (42%) million women perceive no need of using contraceptives for family planning worldwide. Approximately 80 million out of 842 million women use traditional contraceptive methods while 190 million women have the desire to not have pregnancies early in their lives but they do not use any type of contraceptive methods [12]. Some contraceptives including condoms ((21%), withdrawal (5%), female sterilization (24%), male sterilization (2%), and rhythm (3%) methods have been used for decades. Contrary to other methods such as vaginal rings, pills (16%), intrauterine devices (17%), implants (2%), and injectable hormones (8%) are currently available, accessible, and used by the majority of women of reproductive age, especially in Sub-Saharan African regions [11].

Self-efficacy in the right ways of using condoms appears to be effective against the aforementioned health-related problems [13, 14]. However, most women of reproductive age tend not to use them when they are using highly effective contraceptives such as implants, and/or injectable hormones. The prevalence of condom use, for example, varies across national policies, health strategic plans, and/or individual characteristics profiles including age, sex, parity, family-size preferences, awareness, knowledge, peer pressure, sexual masculinity, and lived experiences just to mention a few [15]. Reports have demonstrated that male condoms (21%) and injectable hormones (9.6%) are the dominant used methods by most young people and women of reproductive age respectively.

Despite adherence, the users sometimes compromise the consistency and correct use of male condoms. Appropriate and correct use of condoms may be helping to prevent sexually transmitted infections (STIs) including Human Immunodeficiency Virus (HIV) and unplanned pregnancies by 80-90% [16]. Various organizations have promoted the availability, and accessibility of condoms in public assemblies, marketplaces, religious facilities, playing grounds, nightclubs, dance halls, refugee camps, health facilities, and training institutions/schools [5]. An estimated 4.4 billion out of 13 billion condoms needed yearly in the world are used to curb the prevalence and incidences of STIs/HIV and unplanned pregnancies [11]. Governments, non-governmental agencies, faith-based organizations, and social marketers play a key role in ensuring all people of reproductive age have access to condoms and condom-compatible lubricants [17–19].

Promotion programs of male and female condoms are being implemented on a day-to-day basis through physical contact or media such as radio, television, magazines, social groups, and or publications [4, 20, 21]. However, male condoms appear to have conquered the market, supply, and uptake over female condoms and it may seem unclear whether the demand, availability, accessibility, and uptake of female condoms among women of reproductive health is important to them or not [22]. Despite being the frontline victims of sexual masculinity, the situation is also unknown in Tanzania as to whether the uptake of female condoms among young girls is significantly high or not. Examining female students’ perceived motivators, knowledge, attitudes, and self-reported and intentional female condom practice in training institutions seems to be a key precursor in addressing the challenge.

## Methods and materials

### Study design and study area

An institutional-based analytical cross-sectional design in a quantitative research approach from March to May 2022 was employed to quantify knowledge, attitude, and uptake of female condoms among female students admitted to higher training institutions within Dodoma region, Tanzania. The study was conducted in higher training institutions at a single point in time among consented female students who were reached based on their academic schedule with the primary goal of establishing the association between the parameters under study.

#### Study population

The study recruited 384 randomly selected students admitted to higher training institutions within the Dodoma region, Tanzania. The following procedures were performed to determine the minimum sample size for the study using the formula by Cochran 1977 [23].

1 $$n=\frac{p\left(1-p\right){z}^2}{e^2}$$

Whereas; *n* = a minimum sample size

- p = the population proportion from previous studies (52.0%) [24]
- z^2^ = z-value (1.96) at reliability level (95%) or significance level (5%)
- *e*^2^ = acceptable sampling error (*e* = 0.05)

Thus; $$n=\frac{p\left(1-p\right){z}^2}{e^2}$$ = $$n=\frac{0.438\left(1-0.438\right){1.96}^2}{0.05^2}$$ = $$n=\frac{0.438\left(1-0.438\right)3.8416}{0.0025}$$ = $$n=\frac{0.94563280}{0.0025}$$ = *n* = 383.5

Therefore, the determined minimum sample size in this study was *n* = 384 study participants.

### Sampling procedures

Only consented female students living in-campus and/or off-campus in the respective training institutions within Dodoma region participated in the study. Higher training institutions were selected purposively because there were only two of them in the region, which were then stratified into governmental and private (faith-based) owned respectively. Study respondents were selected by systematic random sampling via a random table number. A list of female students was established from the daily attendance sheets of the respective higher training institutions (*n* = 20,484). Since, 20,484/384 = 53, a 1-in-53 systematic sampling was performed. A random starting point was 3 and using random number tables the procedure continued from that on until a minimum required sample of 384 participants was reached. Institution, program, and year of study then stratified them. As shown in Table 1, the proportionate formula was used to establish a representative sample per stratum.

**Table 1:** Proportional distributions of the study respondents by institution, program, and year of study (*n* = 384)

Strata	Available population (n)	Sampled population ***n*** = [(p_**1 ×**_(n÷p)]
**Institutions**		
I	17,783	333
II	2701	51
**Programs**		
Health Sciences	3926	74
Social Science	3154	59
Humanities	3608	68
Education	4067	76
Informatics and Virtual Sciences	2503	47
Natural and Mathematical sciences	1394	26
Earth sciences	1832	34
Year of Study		
1^st^ yr.	7291	137
2^nd^ yr.	5398	101
3^rd^ yr.	4498	84
4^th^ yr.	3297	62

Source: Study plan (2022)

### Data collection procedure

The principal investigator assisted by the trained research assistants collected data using interviewer-administered structured questionnaires to assess female students’ knowledge, attitude, and self-reported and intentional practices of female condom use. Separate and unoccupied rooms available in the respective training institutions’ premises were used to assure privacy. Respondents were seated in independent chairs to minimize sharing, copying, and pasting of responses from one another. Brief instructions were provided to the respondents before the filling process of questionnaires and the research team was available throughout the process to supervise, respond to queries, collect filled-up questionnaires and secure them. Codes were used instead of respondents’ names in the questionnaires to assure confidentiality. Thirty (30) to forty-five (45) minutes was an approximate time for the completion of filling up the questionnaires.

### Data collection instrument and variable measurements

The research tool for data collection was adopted from previous studies [22, 24–26]. The tools have been currently updated in assessing the uptake of female condoms and females’ knowledge and attitude towards them. To match with the Tanzanian settings the tools were pretested by the principal investigator and scrutinized by statisticians and expert colleagues for language, clarity, and content appropriateness based on the literacy status of the respective study population. It consisted of 34 items in 4 parts including respondents’ socio-demographic characteristics profiles (*n* = 8 items), knowledge about female condoms (*n* = 10 items), female condom attitude (*n* = 10 items), self-reported uptake of female condoms (*n* = 4 items) and the intention to female condom uptake (2 items).

Measurements of variables in this study were informed by previous studies. Knowledge items had “Yes” and “No” responses of which a weight of “1” point was assigned to the “Yes” response indicating the correct response otherwise “0” point to the “No” response indicating the incorrect response. The scores were then computed and a cumulative score of 10 points was established of which a mean score of 6 ± 1.6 was treated as a cut-off point to define the end point of analyzing knowledge. The overall knowledge scores were then transformed into new knowledge categories based on the cut-off point. The highest points for knowledge were defined as adequate knowledge otherwise, not. Attitude items were on 5-point Likert scales ranging from “1” strongly disagree to “5” strongly agree.

For the descriptive purpose, items responses were transformed into quartile measurements including “Agree”, “Neutral” and “Disagree” categories. The highest points were considered “positive attitude”, the median point “Neutral” and the lowest points “Negative attitude on the medical solid waste management. Self-reported intention to uptake of the female condom was measured by the items with “Yes”, I do not remember” and “No” responses. A point of “+ 1” was assigned to the action while a “-1” point to no action or behavioral intention and 0 was assigned to the undefined (“Did not remember”: for the self-reported practices and “Not sure”: for the intentional practice) among female students. Perceiver motivators were measured by 12 items with “Yes” & “No” responses of which a “1” point was assigned to the “Yes” response indicating that a respective motivator was perceived positively (Influenced the uptake of female condoms among female students). Otherwise, a “0” point was assigned for the “No” response, which was defined in this study as the respective motivator was perceived negatively (No influence) over the uptake of female condoms among female students.

### Validity and reliability

Content validity was opted and it was assured in this study by developing items relevant and appropriate research tools, which were then shared with statisticians and expert colleagues for inputs on the content appropriateness, sentence structure, language, and organization. While other things remained unchanged, their responses required research tools to be translated into the Swahili language to blend with the literacy level of the study respondents and improve the clarity, understanding, accuracy, and completeness of the information. The principal investigator to a sample of 30 respondents in an independent geographical location from the sampled study settings and then piloted tools. Observation from a pilot study revealed that all items were appropriate and clear and the questionnaires would be filled and completed within a range of 30 to 60 min. Findings of the pilot study were then subjected to a scale analysis to determine the reliability measure of the tools of which a Cronbach α = 0.70 for knowledge, 0.73 for attitude, self-reported practices = 0.69, and 0.67 for the intentional practices and thus, as recommended by previous scholars [27–29] that a Cronbach alpha of ≥0.7 is considered a strong and reliable tool, the research tools of this study were, therefore, considered reliable for the actual field data collection.

### Data analysis

With the aid of the Statistical Package for Social Sciences computer software program version 25 available in the institution, data were cleaned and analyzed descriptively. Socio-demographic characteristics profiles of the study respondents and the characterization of knowledge, attitude, self-reported and intentional practices of female condom use were analyzed descriptively quantified and presented in frequencies and percentages. The Chi-square test and cross-tabulation analysis established the relationship between variables, while the binary and a multinomial logistic regression model was used to determine the association between predictor variables and the outcomes of interest under study which was set at a 95% confidence interval and 5% significance level.

The following logistic regression model was used

2 $$\left[p=\frac{1}{1+e{-}^{\left({b}_0+{b}_1x\right)}}\right]\ \left(\le 0\ p\le 1\ \right)$$

Whereas; *Ƥ:* predicted probability of an outcome

- *e:* Exponential
- *b*_*0:*_ Constant value
- *b*_*1*_*:* Slope
- *x:* predictor variable

## Results

### Socio-demographic characteristics profiles of female students

Findings in Table 2 show that the response rate was 100% (*n* = 384) and the mean age of study respondents was 24 ± 2.1 years while the prominent age group was 25-34 years (46.1%). The majority of them (71.1%) were living in-campus at the respective training institutions of which 35.7% were in their first year of studies. 98.2% of the study respondents had never attended any training on sexual and reproductive health be it at home or training institutions. Nevertheless, 97.4% have joined peer groups. 28.1% of the respondents engaged in drug abuse and 24.5% had traveled for academic tours. Refer to the table for other findings.

**Table 2:** Socio-demographic characteristics profiles of the study respondents (*n* = 384)

Variables	Frequency (%)
**Residence**	
Institution I	333 (86.7)
Institution II	51 (13.3)
**Program**	
Health Sciences	74 (19.2)
Social Science	59 (15.4)
Humanities	68 (17.7)
Education	76 (19.8)
Informatics and Virtual Sciences	47 (12.2)
Natural and Mathematical sciences	26 (6.8)
Earth sciences	34 (8.9)
**Year of Study**	
1^st^ yr.	137 (35.7)
2^nd^ yr.	101 (26.3)
3^rd^ yr.	84 (21.9)
4^th^ yr.	62 (16.1)
**Age: M = 24 ± 2.12**	
< 18 yrs.	21 (5.5)
19–24 yrs.	156 (40.6)
25–34 yrs.	177 (46.1)
> 35 yrs.	30 (7.8)
**Accommodation**	
In-campus	273 (71.1)
Off-campus	111 (28.9)
**Marital status**	
Single	207 (53.9)
Married	33 (8.6)
Cohabitating	141 (36.7)
Divorced	3 (0.8)
**Religion**	
Muslim	178 (46.5)
Christian	206 (53.5)
**Previous training**	
Yes	7 (1.8)
No	377 (98.2)
**Drug abuse**	
Yes	108 (28.1)
No	276 (71.9)
**Peer groups**	
Yes	374 (97.4)
No	10 (2.6)
**Academic tours**	
Yes	94 (24.5)
No	290 (75.5)

Source: Field data (2022)

### Proportional distribution of self-reported use of female condoms among female students in higher training institutions Dodoma, Tanzania

**Fig. 1:**
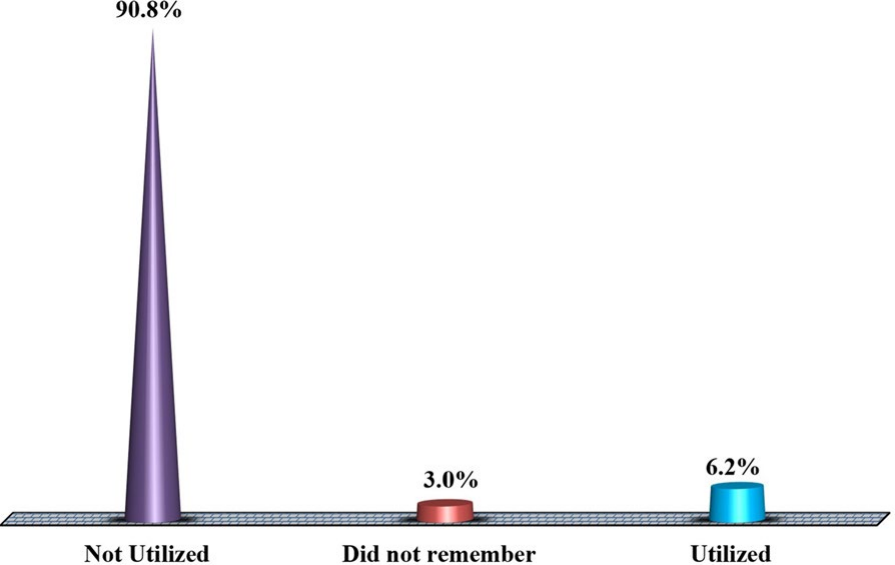
Proportional distribution of self-reported use of female condoms among female students in higher training institutions in Dodoma, Tanzania (*n* = 384). Source: Field data (2022)

Figure 1 presents findings that demonstrate the uptake of female condoms among female students admitted to higher training institutions within Dodoma region, Tanzania. It was revealed that the highest proportion of them (90.8%) have never and 3.0% did not remember whether they used them during sexual intercourse or not. Refer to the figure for other findings.

### Proportional distribution of perceived motivators for female condoms uptake among female students in higher training institutions in Dodoma, Tanzania

Findings in Table 3 show the perceived motivators that would have influenced or hampered the uptake of female condoms among female students in higher training institutions within Dodoma region, Tanzania. The majority (26.3%) of the study respondents reported that they did not even ever see packages of female condoms for them to be motivated to opt to use them. Moreover, 15.2% (*n* = 58), 11.8% (*n* = 45), and 9.4% (*n* = 37) of them reported that societal accusations towards female users of female condoms demotivated them from using them fearing that they would be labeled as promiscuous, infected individuals with STIs/HIV and not faithful to their male sexual collaborates respectively. Refer to the table for other findings.

**Table 3:** Proportional distribution of perceived motivators for female condoms uptake among female students in higher training institutions withinn Dodoma, Tanzania (*n* = 384)

Motivator items	n (%)
Never been exposed to female condom	101 (26.3)
Availability of female condom in resource-centers/boxes as for male condoms	18 (4.7)
Availability and availability in shops	27 (7.1)
Avoidance of being treated as promiscuous	58 (15.2)
Avoidance of being treated as infected individual with STIs/HIV	45 (11.8)
Avoidance of being treated as not faithful	37 (9.4)
Myth that females do not wear condoms during sexual intercourses	24 (6.3)
Big ring sizes than vaginas	23 (5.9)
Not attractive/smell good as some male condoms	21 (5.5)
Cumbersome to carry	14 (3.7)
Awkward to wear and remove	8 (2.1)
Pressure from male sexual partners	8 (2.0)

Source: Field data (2022)

### Proportional distribution of knowledge about female condoms among female students in higher training institutions in Dodoma, Tanzania

**Fig. 2:**
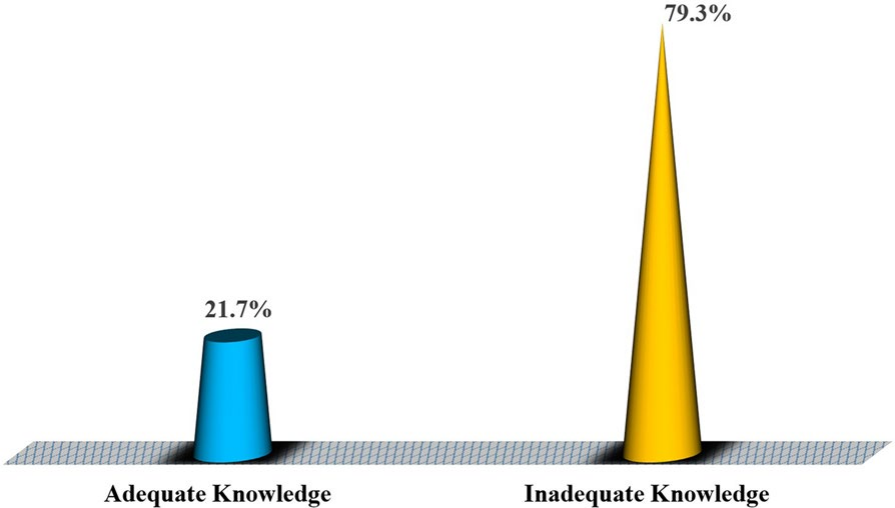
Proportional distribution of knowledge about female condoms among female students in higher training institutions in Dodoma, Tanzania (*n* = 384). Source: Field data (2022)

As shown in Fig. 2, 79.3% (*n* = 305) of the study respondents had inadequate knowledge of female condoms.

### Factors associated with knowledge about female condoms among female students admitted in higher training institutions within Dodoma region, Tanzania

Table 4 shows that several factors [Health sciences programs, year of study (3^rd^ and 4^th^ years), age (25-34 and > 35 years age groups)], living in-campus, living single, previous training, peer groups, and academic tours) were observed to be associated significantly with female students’ knowledge of female condoms. The odds of them having adequate knowledge about it was 17.7% attributed to being enrolled and studying in health science programs (AOR = 1.769; *p* < 0.05; 95%CI: 0.836, 3.240). Female students in 3^rd^ and 4^th^ years of their studies had higher adds of knowing female condoms (AOR = 1.344; *p* < 0.05; 95%CI: 0.645, 2.940) and (AOR = 1.901; *p* < 0.05; 95%CI: 0.934, 3.663) compared to their counterparts 1^st^ and 2^nd^ years students respectively.

**Table 4:** The association between Socio-demographic characteristics profiles and knowledge about female condom among female students admitted in higher training institutions within Dodoma, Tanzania (*n* = 384)

Variables	COR	***P***-value	95%CI		AOR	***P***-value	95%CI	
			Low	Upper			Low	Upper
**Residence**								
Institution I	1.021	0.063	0.852	2.877	0.925	0.902	0.228	1.034
Institution II	1				1			
**Program**								
Health Sciences	2.014	0.016	1.743	4.020	1.769	0.036	0.836	3.240
Social Science	0.532	0.138	0.195	1.271	0.339	0.172	0.147	1.030
Humanities	0.883	0.076	0.502	1.935	0.611	0.139	0.203	1.314
Education	1.034	0.082	0.634	2.731	0.933	0.102	0.530	2.553
Informatics and Virtual Sciences	0.774	0.091	0.348	2.441	0.467	0.162	0.108	1.005
Natural and Mathematical sciences	1.723	0.073	0.872	4.404	1.301	0.112	0.632	3.932
Earth sciences	1				1			
**Year of Study**								
1^st^ yr.	1				1			
2^nd^ yr.	0.772	0.082	0.445	2.067	0.476	0.124	0.103	1.471
3^rd^ yr.	1.843	0.037	0.721	3.329	1.344	0.042	0.645	2.940
4^th^ yr.	2.103	0.022	1.545	4.833	1.901	0.039	0.934	3.663
**Age**								
< 18 yrs.	1				1			
19–24 yrs.	0.553	0.138	0.106	1.068	0.218	1.144	0.025	0.971
25–34 yrs.	2.004	0.019	1.046	5.233	1.663	0.048	0.894	3.205
> 35 yrs.	4.221	0.001	2.011	7.881	3.574	0.015	1.034	5.139
**Accommodation**								
In-campus	3.022	0.001	1.064	7.211	2.422	0.011	1.101	5.290
Off-campus	1							
**Marital status**								
Single	2.472	0.013	1.310	5.116	1.992	0.035	0.820	3.201
Cohabitating	0.962	0.126	0.361	2.454	0.703	0.155	0.321	2.087
Divorced	1.003	0.078	0.903	3.022	0.909	0.134	0.528	2.311
Married	1				1			
**Religion**								
Muslim	1							
Christian	0.754	0.091	0.235	2.042	0.446	0.120	0.185	1.772
**Previous training**								
Yes	7.203	0.001	4.021	11.042	5.307	0.001	2.310	9.209
No	1				1			
**Drug abuse**								
Yes	0.821	0.037	0.452	1.899	0.663	0.103	0.177	1.542
No	1				1			
**Peer groups**								
Yes	3.206	0.002	1.043	7.322	2.990	0.007	1.003	5.094
No	1				1			
**Academic tours**								
Yes	2.118	0.027	1.338	5.204	1.788	0.041	0.768	3.210
No	1							

Source: Field data (2022)

Findings indicate that being at 25-34 years (AOR = 1.663; *p* < 0.05; 95%CI: 0.894, 3.205); > 35 years (AOR = 3.574; *p* < 0.05; 95%CI: 1.034, 5.139) and living single (AOR = 1.992; *p* < 0.05; 95%CI: 0.820, 3.201) were associated significantly with female students’ knowledge about female condoms than others in the respective variables. Nevertheless, findings reveal that the odds of female students having adequate knowledge about female condoms were high among those who resided in-campus (AOR = 2.422; *p* < 0.05; 95%CI: 1.101, 5.290) against those who were living off-campus. Moreover, exposure to previous training about sexual and reproductive health matters and peer groups and academic tours were associated significantly with female students’ adequate knowledge about female condoms (AOR = 5.307; *p* < 0.01; 95%CI: 2.310, 9.209), (AOR = 2.990; *p* < 0.01; 95%CI: 1.003, 5.094) and (AOR = 1.788; *p* < 0.05; 95%CI: 0.768, 3.210) respectively. Refer to the figure for other findings.

### Proportional distribution of attitude about female condoms among female students in higher training institutions Dodoma, Tanzania

**Fig. 3:**
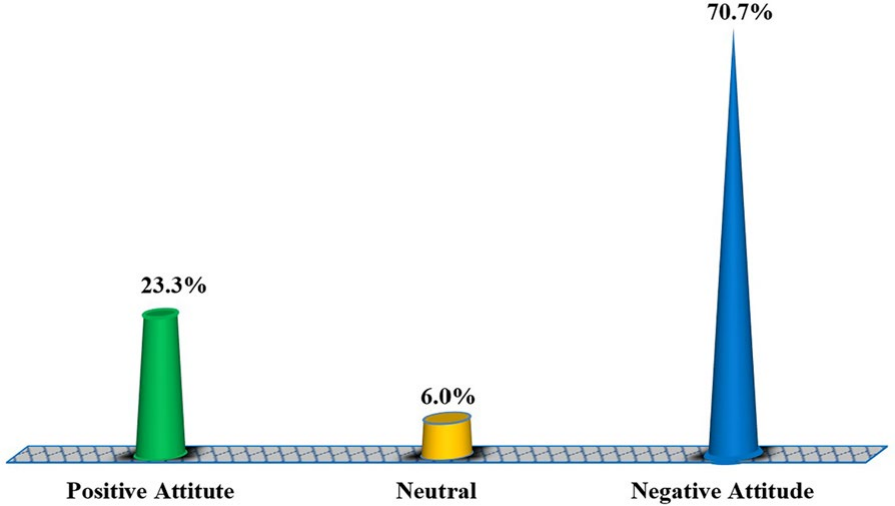
Proportional distribution of attitude about female condoms among female students in higher training institutions in Dodoma, Tanzania (*n* = 384). Source: Field data (2022)

Findings of respondents’ attitudes towards female condoms in Fig. 3 indicate that majority of them (70.7%) had –ve attitude against it while 6.0% (*n* = 23) of the respondents were at the neutral point to whether they needed to use female condoms or not. Refer to the figure for other findings.

### Factors related to attitude towards female condoms among female students admitted in higher training institutions within Dodoma region, Tanzania

Findings in Table 5 indicate that female students in health science programs were 1.105 (AOR) times more likely to have a positive attitude towards female condoms than those in other programs (*p* < 0.05; 95%CI: 0.755, 3.323). Students aged > 35 years and those in their fourth year of studies had significant odds of developing a positive attitude towards female condoms against their counterparts (AOR = 1.010; *p* < 0.05; 95%CI: 0.738, 2.999) and (AOR = 1.012; *p* < 0.05; 95%CI: 0.704, 2.833) respectively. Variables such as living in-campus (AOR = 1.039; *p* < 0.05; 95%CI: 0.348, 2.630); being single (AOR = 1.201; *p* < 0.05; 95%CI: 0.755, 2.401); Previous training about sexual and reproductive health issues (AOR = 2.121; *p* < 0.05; 1.420, 4.335); exposure to peer groups (AOR = 1.041; *p* < 0.05; 95%CI: 0.674, 2.005) and adequate knowledge (AOR = 1.108; *p* < 0.05; 95%CI: 0.543, 2.794) were the positive predictor variables of female students’ positive attitude towards female condoms. Refer to the figure for other findings.

**Table 5:** The association between Socio-demographic characteristics profiles and attitude towards female condom among female students admitted in higher training institutions within Dodoma, Tanzania (*n* = 384)

Variables	COR	***P***-value	95%CI		AOR	***P***-value	95%CI	
			Low	Upper			Low	Upper
**Residence**								
Institution I	0.734	0.057	0.152	1.457	0.552	0.141	0.199	1.709
Institution II	1				1			
**Program**								
Health Sciences	1. 401	0.033	0.347	3.014	1.105	0.042	0.755	3.323
Social Science	0.803	0.068	0.248	1.544	0.430	0.176	0.109	1.845
Humanities	0.720	0.067	0.399	1.831	0.434	0.129	0.104	1.064
Education	0.632	0.071	0.120	1.662	0.221	0.127	0.728	1.397
Informatics and Virtual Sciences	0.892	0.053	0.201	1.840	0.520	0.107	0.115	1.702
Natural and Mathematical sciences	0.605	0.086	0.207	1.709	0.904	0.148	0.304	2.007
Earth sciences	1				1			
**Year of Study**								
1^st^ yr.	1				1			
2^nd^ yr.	0.609	0.078	0.191	1.565	0.207	0.108	0.095	1.047
3^rd^ yr.	0.771	0.090	0.201	1.740	0.458	0.117	0.088	1.042
4^th^ yr.	1.139	0.041	0.601	2.045	1.012	0.048	0.704	2.833
**Age**								
< 18 yrs.	1				1			
19–24 yrs.	1.067	0.081	0.721	3.121	0.860	0.137	0.208	2.016
25–34 yrs.	0.847	0.029	0.299	1.652	0.551	0.223	0.110	1.663
> 35 yrs.	1.673	0.027	0.882	3.111	1.010	0.042	0.738	2.999
**Accommodation**								
In-campus	1.741	0.030	0.892	2.051	1.039	0.043	0.348	2.630
Off-campus	1							
**Marital status**								
Single	1.651	0.030	0.739	3.033	1.201	0.045	0.755	2.410
Cohabitating	1.009	0.066	0.679	2.922	0.907	0.106	0.381	2.056
Divorced	0.567	0.012	0.107	1.777	0.309	0.067	0.015	0.891
Married	1				1			
**Religion**								
Muslim	1							
Christian	1.693	0.024	0.771	2.108	1.702	0.038	0.670	3.603
**Previous training**								
Yes	3.012	0.027	1.902	5.104	2.121	0.036	1.420	4.335
No	1				1			
**Drug abuse**								
Yes	1.801	0.084	0.703	2.102	0.761	0.122	0.203	1.971
No	1				1			
**Peer groups**								
Yes	1.505	0.032	0.792	2.102	1.041	0.044	0.674	2.005
No	1				1			
**Academic tours**								
Yes	0.765	0.081	0.107	2.011	0.501	0.104	0.206	1.110
No	1							
**Knowledge**								
Adequate	1.885	0.029	0.743	3.218	1.108	0.040	0.543	2.794
Inadequate	1				1			

Source: Field data (2022)

### Proportional distribution of intentional practice of female condom use among female students in higher training institutions Dodoma, Tanzania

**Fig. 4:**
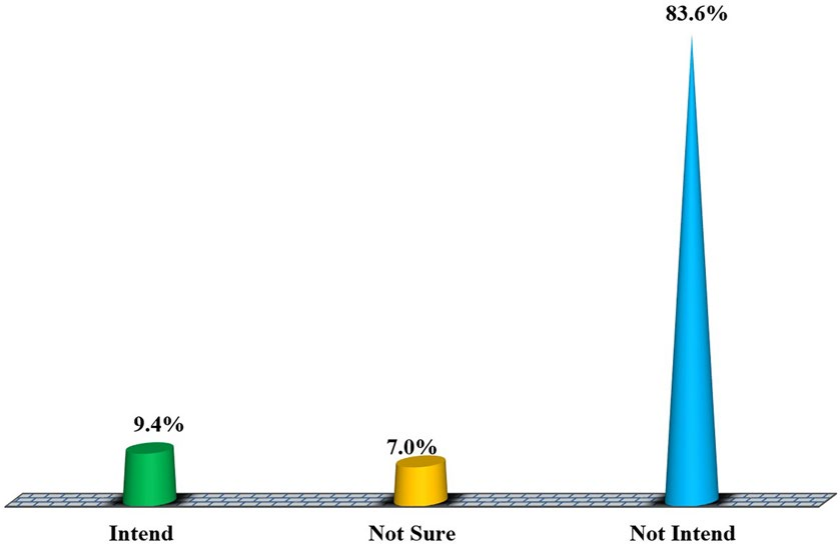
Proportional distribution of intentional practice of female condom use among female students in higher training institutions in Dodoma, Tanzania. Source: Field data (2022)

Findings in Fig. 4 show proportional distributions of the study respondents’ intentions to use female condoms during their next sexual intercourse. 83.6% of them did not report an intention that they would use female condoms when having sexual intercourse with their partners. Moreover, 7.0% of the respondents were not sure whether they would use female condoms during sexual intercourse or not. Refer to the figure for other findings.

### Factors related to intentional uptake of female condoms among female students admitted in higher training institutions within Dodoma region, Tanzania

As depicted in Table 6, female students in the institution I (faith-based higher training institution) were 1.018 (AOR) times more likely to uptake female condoms than their counterparts in institution II (Governmental based higher training institution: *p* < 0.05; 95%CI: 0.232; 2). Additionally, the odds of female students uptake female condoms were significantly associated with studying in health science programs (AOR = 1.105; *p* < 0.05; 95%CI: 0.755, 3.324); being at 2^nd^ year of studies (AOR = 1.539; *p* < 0.05; 95%CI: 0.973, 3.307); 3^rd^ year of studies (AOR = 1.046; *p* < 0.05; 95%CI: 0.751, 2.602); < 18 years of age (AOR = 1.430; *p* < 0.05; 95%CI: 0.546, 3.016) and being at 19-24 years age group (AOR = 1.032; *p* < 0.05; 95%CI: 0.865, 2.2.313).

**Table 6:** The association between Socio-demographic characteristics profiles and intentional practice of female condom among female students admitted in higher training institutions within Dodoma, Tanzania (*n* = 384)

Variables	COR	***P***-value	95%CI		AOR	***P***-value	95%CI	
			Low	Upper			Low	Upper
**Residence**								
Institution I	1.464	0.023	0.402	2.505	1.018	0.047	0.232	2.105
Institution II	1				1			
**Program**								
Health Sciences	1.421	0.033	0.347	3.014	1.105	0.042	0.755	3.324
Social Science	1.239	0.071	0.602	2.632	1.004	0.108	0.330	2.201
Humanities	0.822	0.077	0.442	2.431	0.798	0.106	0.236	1.965
Education	1.064	0.092	0.604	2.666	0.886	0.104	0.433	1.898
Informatics and Virtual Sciences	0.702	0.088	0.309	1.922	0.409	0.171	0.021	1.541
Natural and Mathematical sciences	0.830	0.065	0.510	1.990	0.582	0.122	0.106	1.844
Earth sciences	1				1			
**Year of Study**								
1^st^ yr.	1				1			
2^nd^ yr.	2.237	0.001	1.404	5.212	1.559	0.025	0.973	3.307
3^rd^ yr.	1.867	0.028	0.837	2.974	1.046	0.031	0.751	2.602
4^th^ yr.	0.678	0.071	0.202	1.892	0.487	0.104	0.130	1.643
**Age**								
< 18 yrs.	1				1			
19–24 yrs.	2.301	0.010	1.048	5.201	1.430	0.021	0.546	3.016
25–34 yrs.	1.337	0.034	0.877	3.129	1.032	0.042	0.865	2.313
> 35 yrs.	0.955	0.693	0.432	2.783	0.712	0.107	0.463	1.088
**Accommodation**								
In-campus	0.871	0.073	0.304	1.894	0.648	0.116	0.105	1.974
Off-campus	1							
**Marital status**								
Single	2.109	0.039	1.667	5.211	1.746	0.046	0.503	3.104
Cohabitating	0.801	0.072	0.555	1.721	0.674	0.230	0.222	1.533
Divorced	1.562	0.901	0.642	2.522	1.272	0.104	0.566	2.322
Married	1				1			
**Religion**								
Muslim	1							
Christian	0.688	0.065	0.202	1.078	0.405	0.107	0.107	1.535
**Previous training**								
Yes	2.244	0.008	1.320	5.661	1.949	0.021	0.855	3.540
No	1				1			
**Drug abuse**								
Yes	0.435	0.092	0.104	1.687	0.303	0.122	0.034	1.855
No	1				1			
**Peer groups**								
Yes	2.115	0.023	1.450	4.789	1.897	0.047	0.543	3.223
No	1				1			
**Academic tours**								
Yes	0.868	0.070	0.632	1.348	0.775	0.104	0.544	1.885
No	1							
**Knowledge**								
Adequate	2.446	0.011	1.940	4.444	1.343	0.031	0.848	3.202
Inadequate	1				1			
**Attitude**								
+ve	1.433	0.009	0.784	3.233	1.039	0.035	0.774	2.560
-ve								

Source: Field data (2022)

Findings in the table demonstrate that the odds of being single (AOR = 1.746; *p* < 0.05; 95%CI: 0.503, 3.104); exposure to previous training on sexual and reproductive health matters (AOR = 1.949; *p* < 0.05; 95%CI: 0.855, 3.540); exposure to peer groups (AOR = 1.897; *p* < 0.05; 95%CI: 0.543, 3.223); adequate knowledge (AOR = 1.343; *p* < 0.05; 95%CI: 0.848, 3.202) and having a positive attitude (AOR = 1.039; *p* < 0.05; 95%CI: 0.774, 2.560) impacted significantly female students’ intentional uptake of female condoms. Refer to the figure for other findings.

## Discussion

The study found that the uptake and intention to use female condoms among female students who were admitted to higher training institutions within Dodoma region, Tanzania was significantly low. The majority of them had never seen even a single package of female condoms for them to be convinced to use while some did not dare use them because they were afraid to be treated as promiscuous, infected individuals with STIs/HIV or not being faithful to their male sexual partners. Although very few, others reported that they did not use female condoms because they have big ring sizes than vaginas, they were not attractive or smell good as male condoms do, cumbersome to carry, awkward to wear or remove after sexual intercourse, and or their male sexual partners did not want them to use condoms.

However, those with adequate knowledge about it, positive attitudes towards them, and those who stayed off-campus and had previous training on sexual and reproductive matters appeared to be the most users of female condoms than others. It may seem to be so obvious because having to know something and develop a positive attitude towards it catalyzes someone to make an informed decision and reasoning to use/consume the product. Moreover, exposure to training and having the widest range possible to interact with peers influences, partners’ pressure, interactive communications, and the diffusion of new knowledge from experts and the environment at large would maximize or minimize the uptake and/or intentions for condom use among female students in higher training institutions within the region.

Findings of the uptake and/or intention to use female condoms are in line with the findings of the descriptive cross-sectional survey found by Ananga et al. [25] on the Knowledge, acceptance, and utilization of female condoms among women of reproductive age in Ghana, which revealed that the female condom knowledge, acceptance, and utilization were significantly low. Friends, media, public lectures, and limited accessibility from shops and health centers were found to be the predictors associated with the findings in their study. Moreover, tallying with the findings of this study, the descriptive study by Pablo et al. [30] on the Spanish validation of female condom attitude scale and female condom use among young women in Colombia, revealed that the utilization of female condoms among women was low while the minority who used them were influenced by peer pressure, sexual partners and or public training.

Needless to say, similarly to a systematic review scholarly work by Fasehun et al.*,* [20] on the barriers and facilitators to the acceptability of female condoms in Low and Middle-income countries unfolded that the uptake of female condoms was significantly low with partners’ acceptability, accessibility, knowledge, and attitude being the prominent determinants of the uptake. Additionally, a quantitative descriptive study by Mokgetse and Ramukumba [26] on female condom acceptability and use amongst young women in Botswana highlighted that there was remarkably low use of female condoms among women regardless of their being aware of them. The similarities of the findings between the previous scholarly works and the study on hand may imply that the problem of low female condom uptake is a global challenge, especially in low and middle-income countries including Tanzania.

Despite low uptake and intentions for female condom use, female majority of female students in this study demonstrated low knowledge about it. The main sources of knowledge were identified to be health facilities, peer groups, media, public campaigns, and training on sexual and reproductive health matters. The minority were able to answer correctly that female condoms can prevent both STIs/HIV, and unplanned pregnancies, they cannot be re-used during sexual intercourse, females can make decisions to overuse them and they are easy to use as male condoms. Findings may imply that despite the existing health-related programs by the government, non-government organizations, and or private sectors, female youths do not know that female condoms are very easy and safe to use and are there for them against STIs/HIV and unplanned pregnancies as male condoms can do.

In support of the findings observed by Ananga et al. [25] as noted above and a cross-sectional study by Uchendu et al. [31] on the awareness and utilization of female condoms amongst youth in Nigeria revealed that the majority of their study respondents were not aware of female condoms while only a minority of them had ever seen them. However, contrary to the findings of this study (probably due to differences in educational systems between the two countries and levels of training institutions), a cross-section survey by Oke et al. [24] on the understanding, of female condoms, their acceptability, accessibility, awareness and knowledge among female public health students in Nigeria found that majority of public health students were aware of female condoms while 22.4% of them had ever seen packs of female condoms.

In line with the quality, scholarly work by Gambir et al. [32] on the opportunities and challenges for the introduction of female condoms among young people in Zambia revealed that very few participants acknowledge female condoms due to misconceptions about their safety, how correct would they use them, availability, affordability of the condoms to them, and the limited power they had over the decisions on sexual activities. Nevertheless, the descriptive qualitative findings observed by Davids et al. [33] on condom use decision-making among adolescents in South Africa depicted that male condoms were more promoted and distributed than female condoms, which again may be linked to the low use of female condoms and that sexual masculinity over sexual intercourses still prevails among young people.

Regarding attitudes towards female condoms among female students, the findings of this study showed that the majority of them had negative perspectives on them. They believed that using them was against their religion while others hold a belief that male condoms are better than female condoms. The situation would be possible probably due to low advocacy, promotion, and distribution strategies of female condoms through health policies, market streams, and/or few female condoms ambassadors as it happens in male condoms. The availability and accessibility of male condoms through automated condoms banks, machines, or banks, would make young females believe and get conditioned that the only trusted and safe condoms to be used during sexual intercourse were male condoms and not otherwise.

Negative attitude towards female condoms among women was also revealed in the scholarly published findings from a descriptive cross-section survey by Obembe et al. [34] on the perceived confidence to use female condoms among tertiary training institutions’ students in Nigeria. They observed that the majority of the study respondents demonstrated low confidence about using female condoms which were attributed to their ethnicities, geographical locations, and negative perceptions of them. Nevertheless, the qualitative exploratory study conducted by Dlamini and Shongwe [35] on the barriers to female condom use among unmarried undergraduate students in Eswatini revealed similar findings to the findings of this study. They found that many undergraduate health sciences students had a negative attitude towards female condoms believing that they were bigger than vaginas, they hinder sexual pleasure, and inadequate knowledge about them.

However, the findings of this study differ from those found by Mantell et al. [36] in their randomized trial design on the perceived male partner attitude toward the female condom in South Africa, which indicated that young especially male partners perceived female condoms positively as male condoms. However, the attributable factors to the mismatch are probably differences in the methodological approaches including study designs, timing, and populations.

## Conclusion

Based on the findings observed in this study it is clear that female students admitted to higher training institutions had low self-reported and intentional practices of female condom use. The trend was significantly linked in this study with their perceptions of the motivators that hindered them to use female condoms and some sociodemographic characteristics profiles, inadequate knowledge, and negative attitude they had toward female condoms. Their accommodation statuses, engagement in peer groups, drug abuse, exposure to previous training on sexual and reproductive health, and marital status were significantly related to their self-reported and intentional practice of using female condoms. Findings may imply that there is a mismatch between the promotion and distribution programs of female over male condoms in higher training institutions be it via health policies, health strategic plans, sexual and reproductive health campaigns or involvement of women of reproductive age in contraceptives use educational interventions. Institutionalized educational programs on sexual and reproductive health matters may need to be given weight and priority to increase the availability, accessibility, and uptake of female condoms among female students.

## Strength of the study

This study addressed the family planning domain in health, which is a very important issue of public concern against not only STIs/HIV but also unplanned pregnancies among young people in response to the call by SDG3. The findings of this study have been established to demonstrate a causal relationship between the variables under study.

## Implications for practices and future research

Policymakers and training institutions, health facility administrative organs in Tanzania, and the globe at large can use the findings from this study to develop innovative strategies for involving and empowering female students with knowledge and attitude to increase the uptake of female condoms as it is done for male condoms. If published in different scientific journals, the findings of this study will provide a relevant base of data and information regarding knowledge, attitude, self-reported and the intentional practice of female students admitted to higher training institutions for large-scale interventions or future research.

## Limitations of the study

The study was conducted in a confined locality and thus findings may not be generalized to care female students of other geographical locations in or outside the country other than those residing in Dodoma region, the central part of Tanzania. The study moreover, did not use a triangulation approach for data collection and thus, the rigor of dependability, transferability, and or confirmability may have not been addressed in this study. Nevertheless, the findings of this study may need to be interpreted with caution, as female students would have faced recall problems to remember and sharing their previous lived experiences about female condom use. Having an opportunity to rate oneself is criticized as it may influence someone to under or overrate or report the habits/information of female students who participated in the study. Therefore, attention may be needed when interpreting the findings of this study.

## Data Availability

Data will be available under special request at walter.millanzi@udom.ac.tz or wcleo87@gmail.com.

## Abbreviations

AOR: Adjusted Odds Ratio
CI: Confidence Interval
COR: Crude Odds Ratio
FC: Female Condom
HIV: Human Immunodeficiency Virus
PhD: Doctor of Philosophy
*p*-value: Probability Value
SDG: Sustainable Development Goals
SPSS: Statistical Package for Social Sciences
STIs: Sexually Transmitted Infections
UNAIDS: United Nations Programme on HIV/AIDS
UNICEF: United Nations Children’s Fund
WHO: World Health Organization
*X*^*2*^: Chi-Square
